# Supplementary figures and images for: Genome-wide analysis identifies Homothorax and Extradenticle as regulators of insulin in Drosophila Insulin-Producing cells
Source: PLoS Genet. 2022 Sep 12;18(9):e1010380. doi: 10.1371/journal.pgen.1010380 (PMC9499297; doi:10.1371/journal.pgen.1010380)

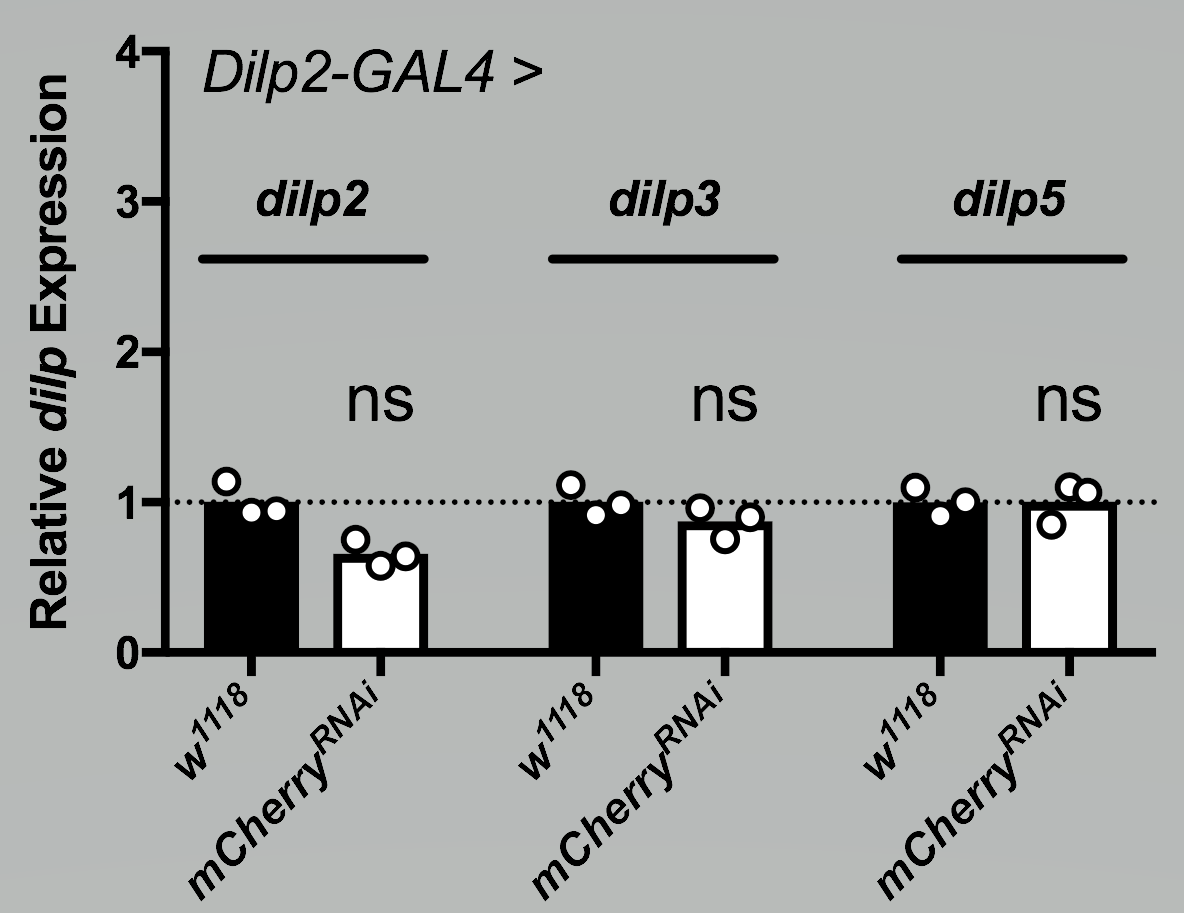

Supplement: S1 Fig — dilp2, -3 and -5 mRNA levels are not significantly different in Dilp2-GAL4R > mCherryRNAi compared to Dilp2-GAL4R /+ fly heads (unpaired two tailed Student’s t-test p > 0.05). (TIFF) [file pgen.1010380.s004.tiff]

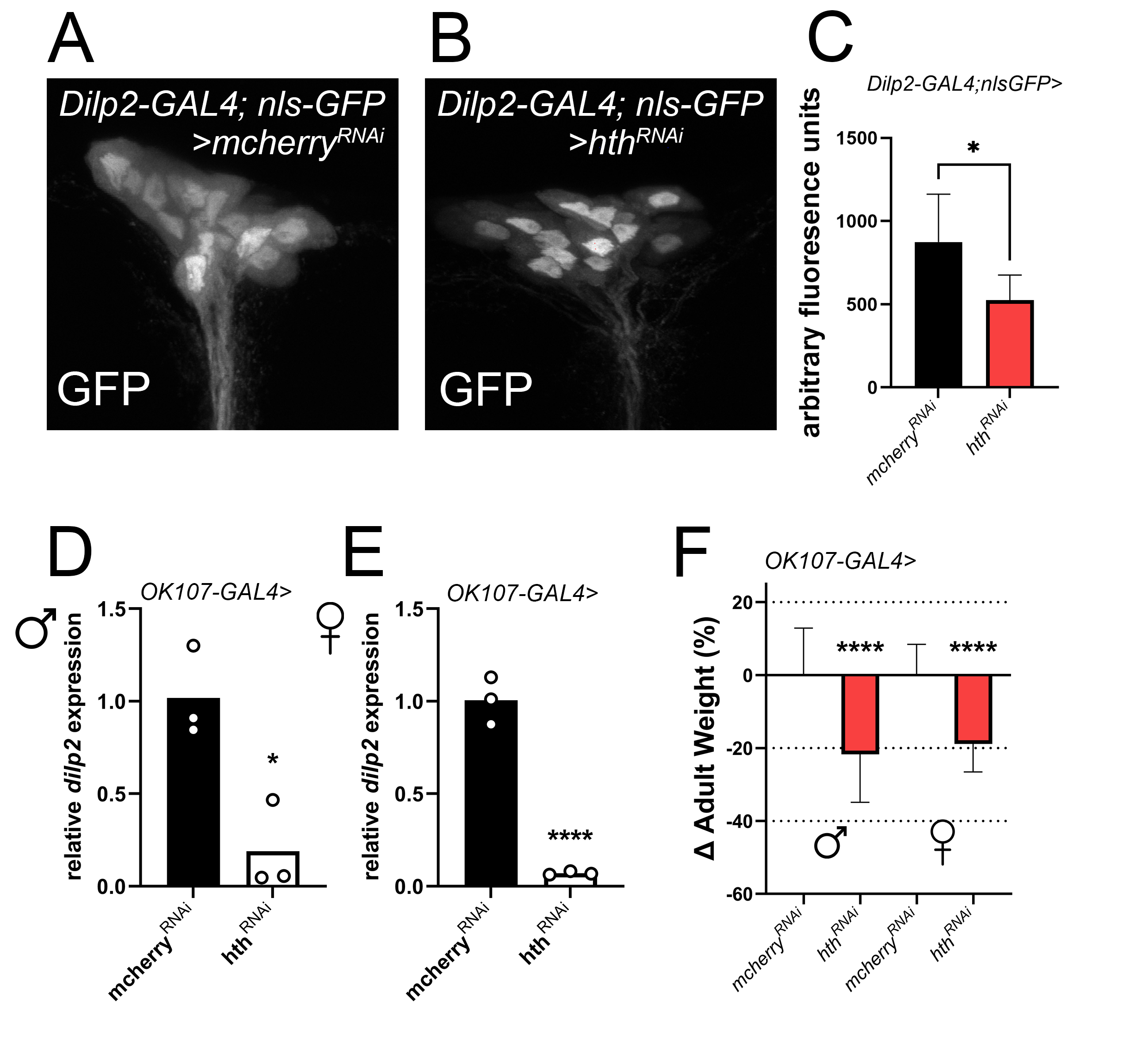

Supplement: S2 Fig — Representative images of adult female IPCs upon knockdown of mcherry (A) or Hth (B), IPCs are visualised by nuclear-GFP (nls-GFP) expression. Images were taken at identical confocal settings. (C) GFP fluorescence in the IPCs is significantly but mildly reduced upon hth depletion (Student’s t-test, * p<0.05). Male (D) and female (E) dilp2 expression is significantly reduced in OK107-GAL4>hthRNAi flies compared to flies expressing an mcherryRNAi control (Student’s t-test, * p<0.05, **** p<0.0001). Adult weight is significantly reduced in OK107-GAL4>hthRNAi male and female flies (Student’s t-test, **** p<0.0001). (TIFF) [file pgen.1010380.s005.tiff]

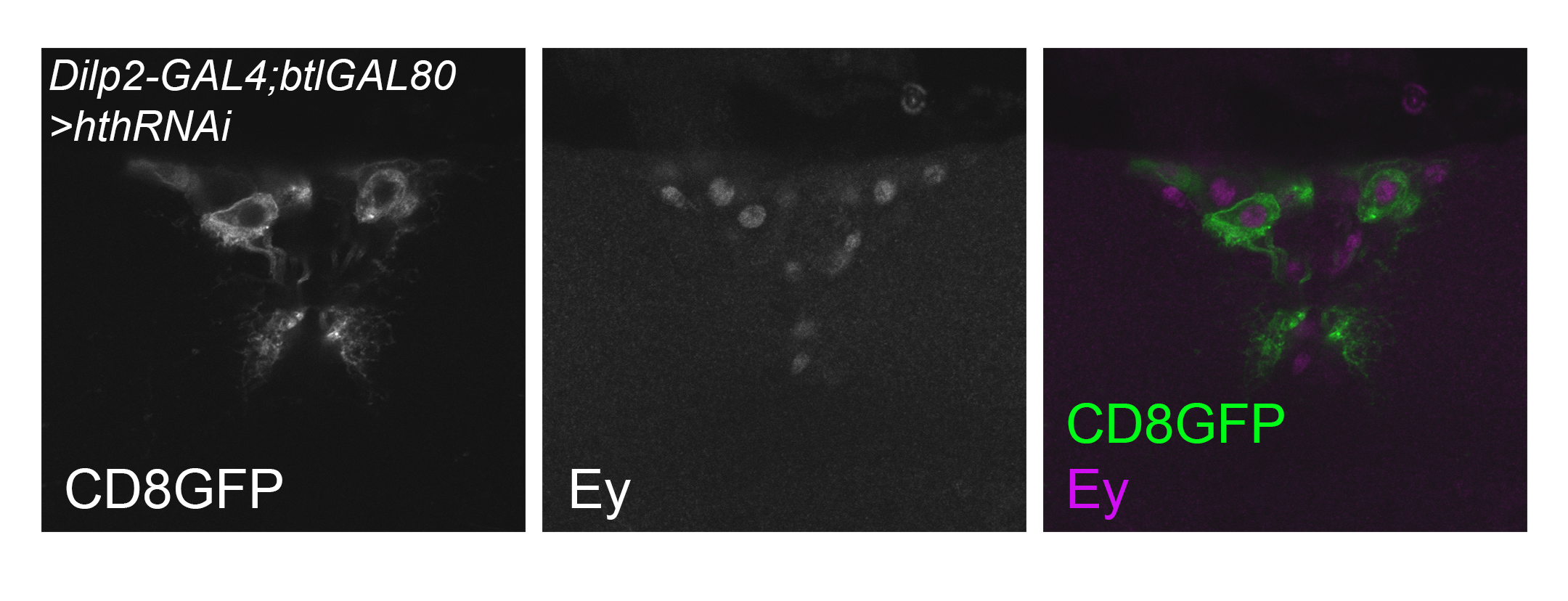

Supplement: S3 Fig — Genetic depletion of hth in the IPCs does not affect Ey expression in the IPCs. IPCs are marked with CD8-GFP driven by Dilp2-GAL4. (TIFF) [file pgen.1010380.s006.tiff]

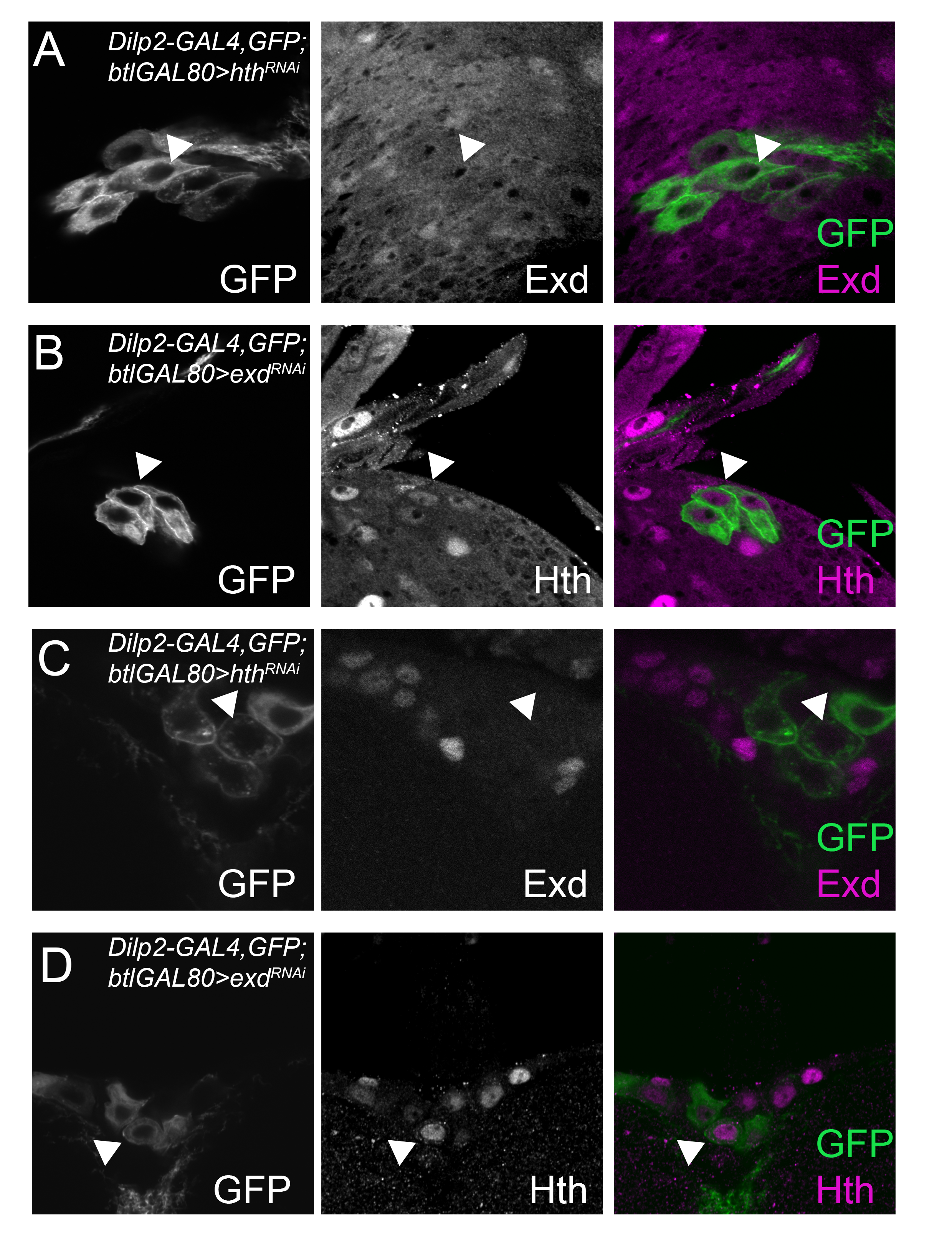

Supplement: S4 Fig — (A) Knockdown of hth results in the absence of Exd in larval IPC nuclei (arrow). (B) Knockdown of exd in larval IPCs does not affect Hth localization. (C) IPC-specific knockdown of hth results in the absence of Exd in IPC nuclei. (D) Hth localization is unaffected upon exd knockdown. (TIFF) [file pgen.1010380.s007.tiff]

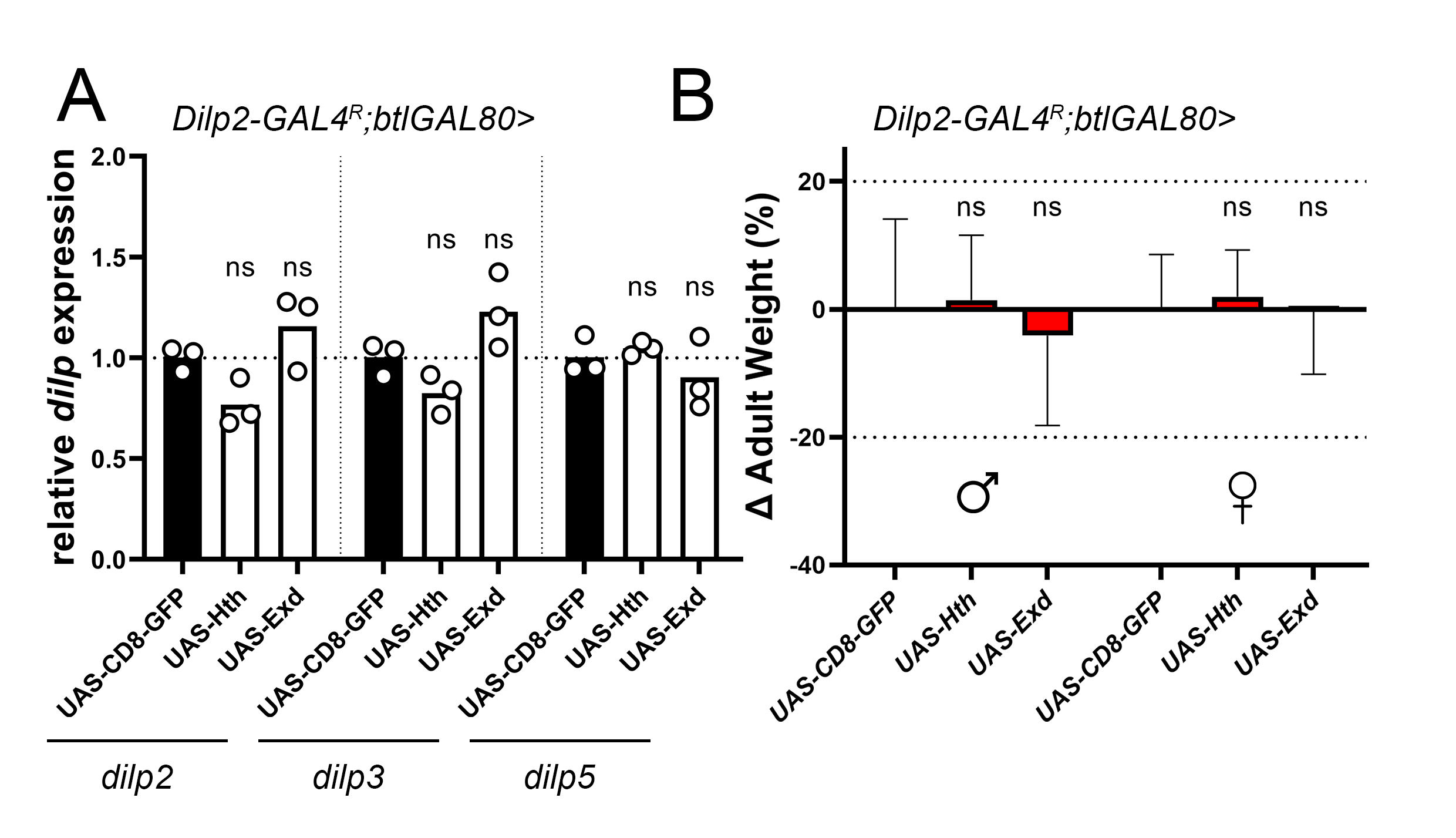

Supplement: S5 Fig — (A) Male or female flies overexpressing Hth or Exd in the IPCs do not weigh significantly more or less than control flies overexpressing GFP. (B) Overexpression of Hth or Exd does not alter dilp2, -3 or -5 mRNA levels (ns, not significant, one-way ANOVA with post-hoc Dunnett’s test to correct for multiple comparisons). (TIFF) [file pgen.1010380.s008.tiff]

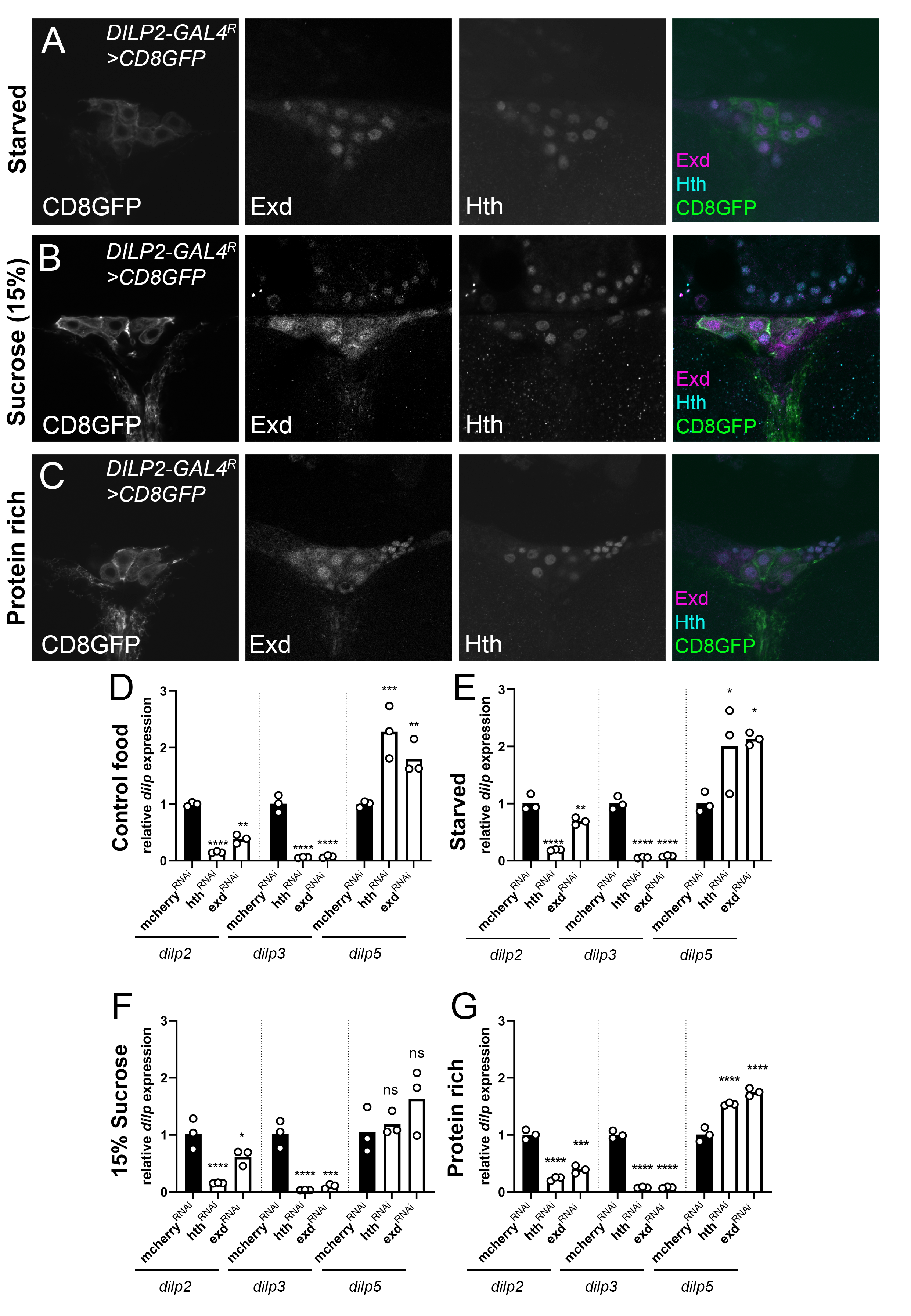

Supplement: S6 Fig — Nuclear localization of Hth and Exd in the IPCs is unaltered in starved (A), sucrose only (B) and protein rich (C) conditions. Flies were reared on control food and 24–48-hour old females were placed on different diets for 72 hours and subsequently dissected and stained. Female flies expressing hthRNAi or exdRNAi driven by dilp2-GAL4R;btlGAL80 were subjected to the same conditions as described above and collected for qPCR. Hth and Exd depletion had identical effects on dilp2, -3 and -5 expression levels in starved (E), sucrose only (F) and protein rich (G) as in control food (D). (ns, not significant, * p < 0.05, ** p <0.01, *** p<0.001, **** p < 0.0001, Dunnett’s multiple comparisons). (TIFF) [file pgen.1010380.s009.tiff]

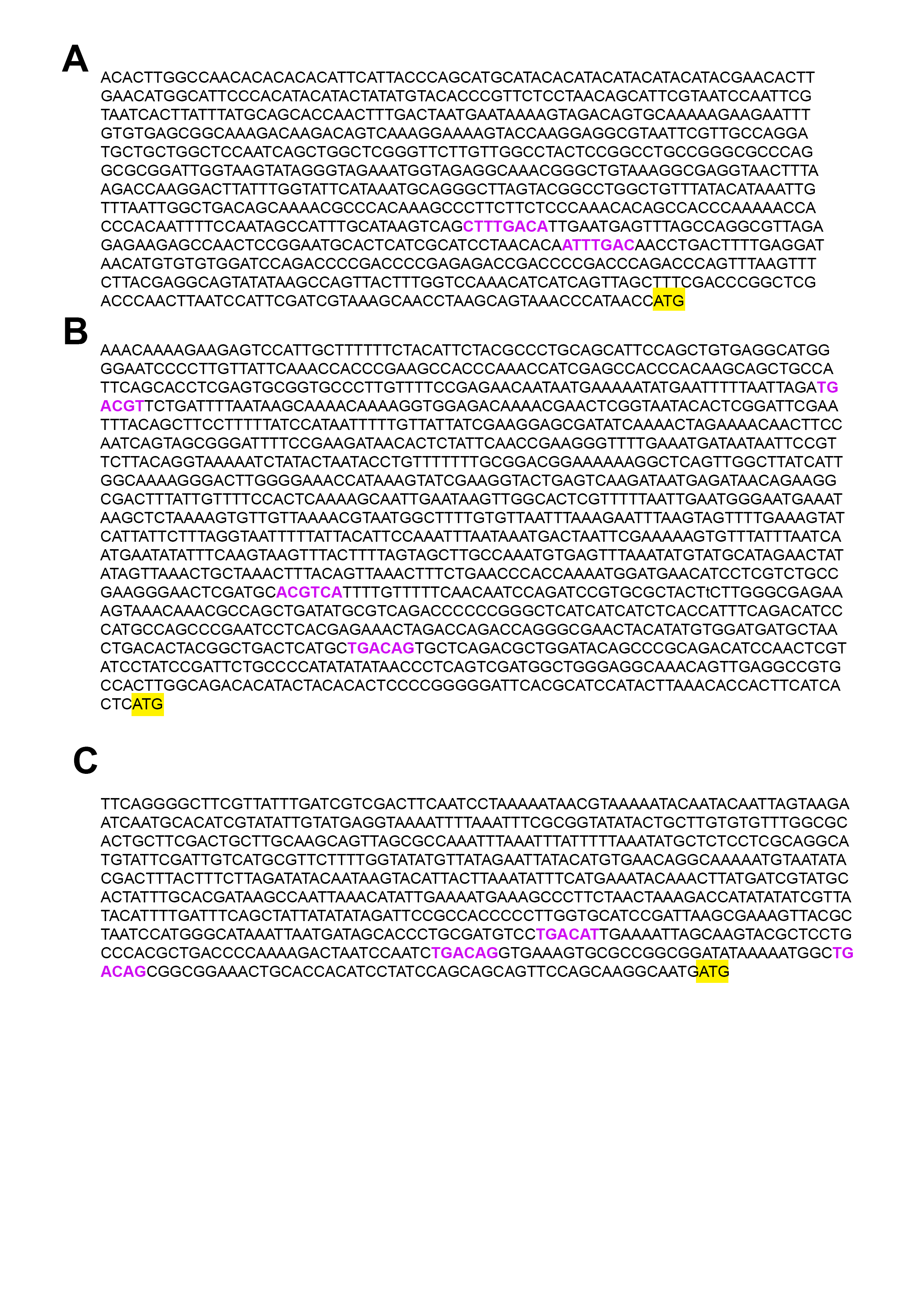

Supplement: S7 Fig — 2, 3 and 3 Hth motifs (purple) were identified in dilp2, -3 and -5 enhancers, respectively. Hth binding motif from the JASPAR database was used to scan in dilp2, -3 and -5 enhancers using the FIMO from the MEME suite (p<0.001, both strands). Start codon is highlighted in yellow. (TIFF) [file pgen.1010380.s010.tiff]

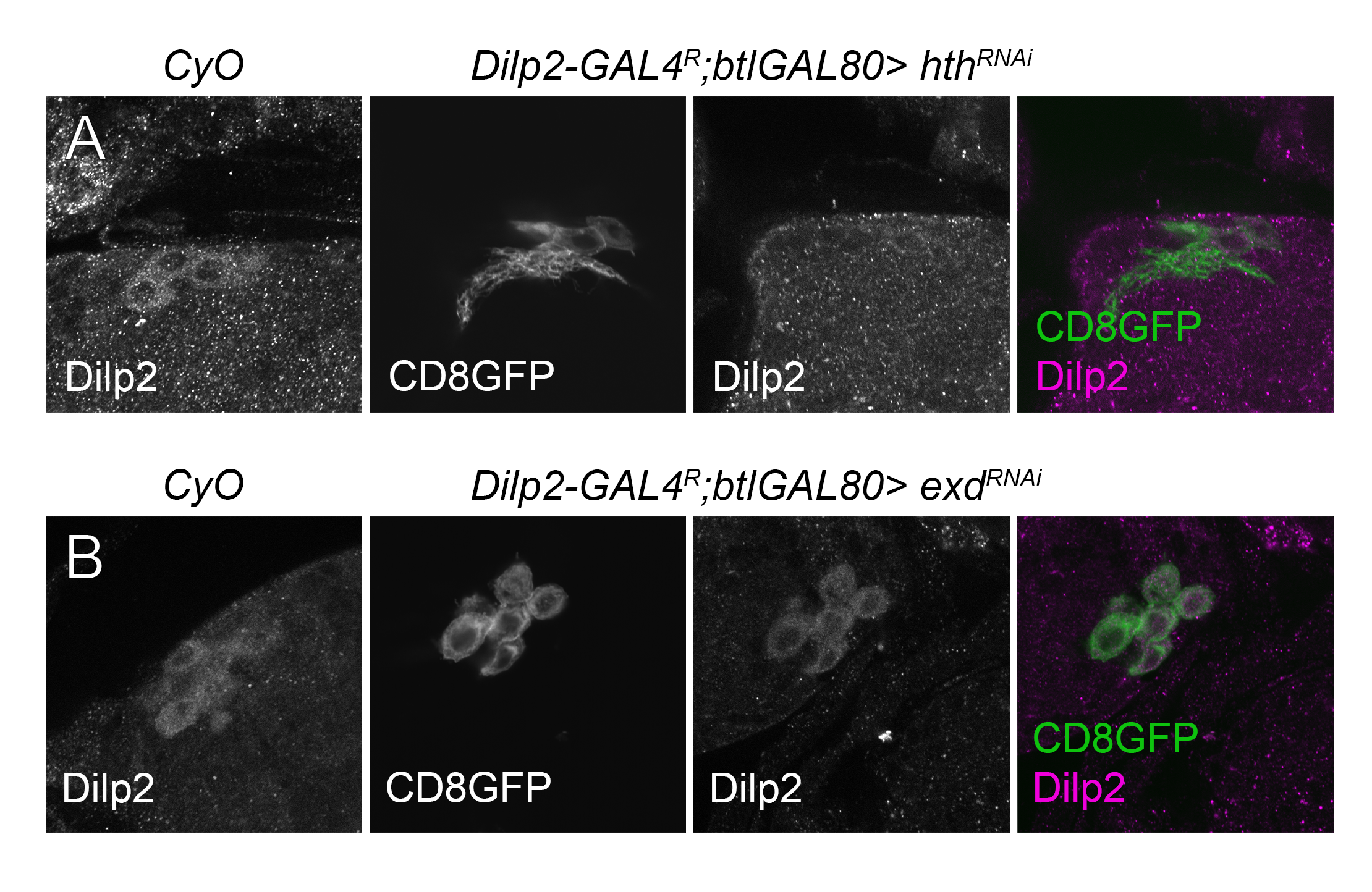

Supplement: S8 Fig — (A-B) Representative images of Dilp2 protein levels in Dilp2-GAL4;btlGAL80> hthRNAi and Dilp2-GAL4;btlGAL80> exdRNAi compared to control siblings (Cyo, left) reared in identical conditions. Flies were reared and age-controlled then brains from knockdown and balanced sibling flies were dissected and stained in the same tube. Expression of CD8GFP was used to differentiate control and test genotypes, and to label the IPCs. Images were acquired using identical confocal settings. (TIFF) [file pgen.1010380.s011.tiff]

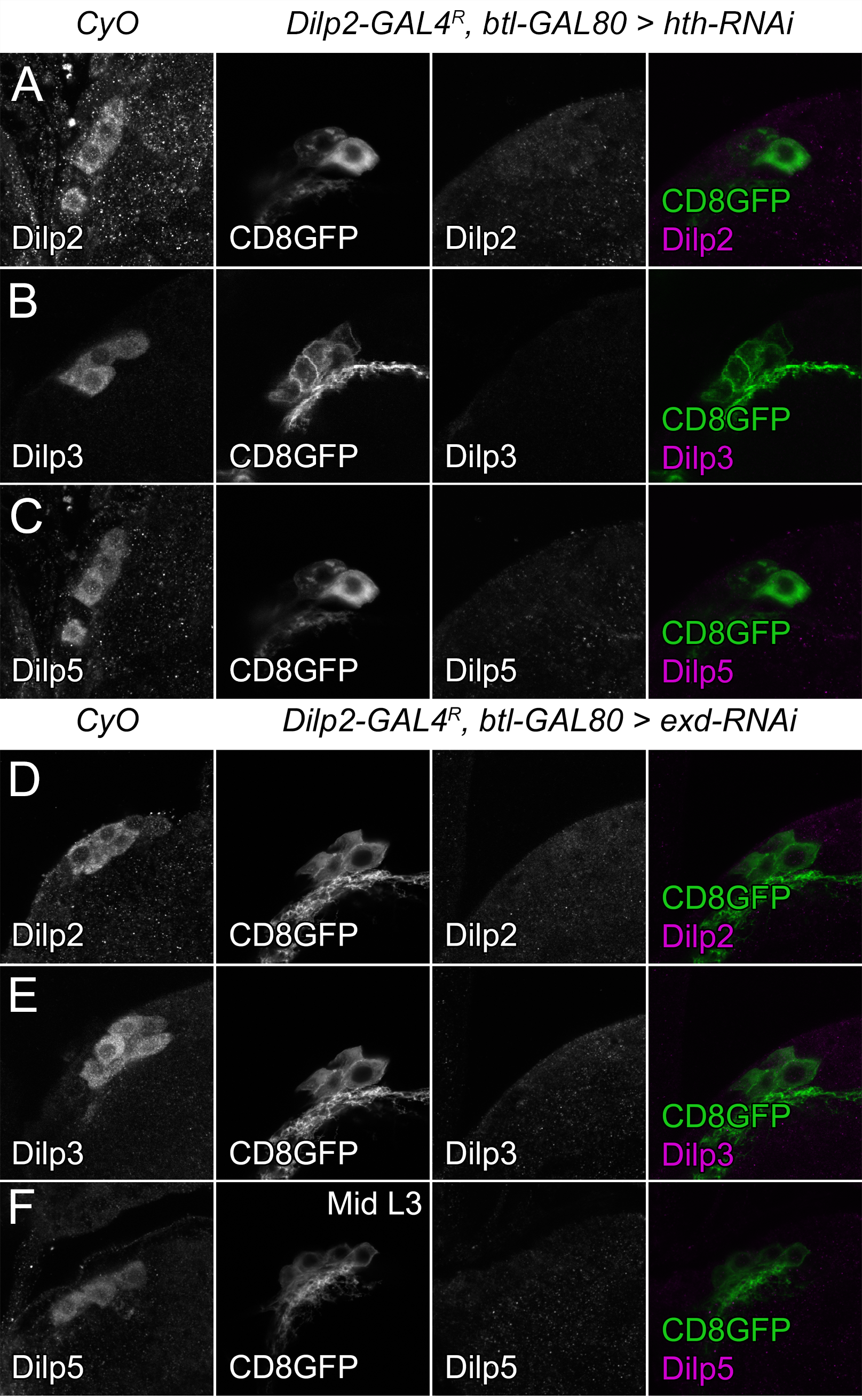

Supplement: S9 Fig — Representative images of Dilp2, 3 and -5 protein levels in (A-C) Dilp2-GAL4;btlGAL80> hthRNAi and (D-F) Dilp2-GAL4;btlGAL80> exdRNAi compared to control siblings (Cyo, left) reared in identical conditions. Flies were reared and age-controlled. Brains from knockdown and balanced sibling flies were dissected and stained in the same tube. Expression of CD8GFP was used to differentiate control and test genotypes, and label IPCs. Images were acquired using identical confocal settings. (TIFF) [file pgen.1010380.s012.tiff]

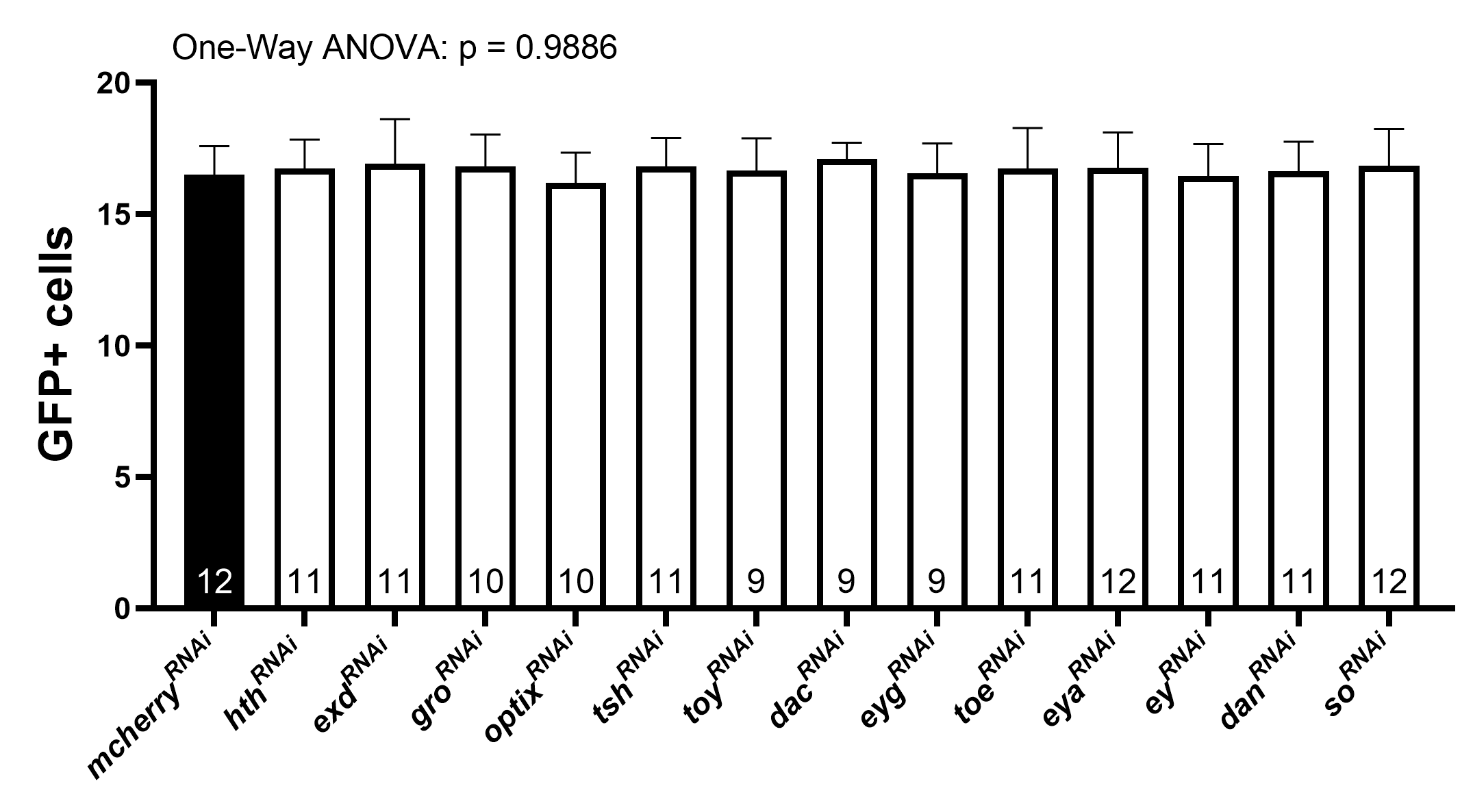

Supplement: S10 Fig — Female flies expressing RNAis against RDGN transcription factors using Dilp2-GAL4;nls-GFP were dissected 4–6 days post-eclosion, fixed and mounted. The number of IPCs was determined by manually counting GFP+ nuclei. Numbers of brains analysed per genotype are shown inside the bars (ns, not significant, one-way ANOVA with post-hoc Dunnett’s test to correct for multiple comparisons). (TIFF) [file pgen.1010380.s013.tiff]
