# Supplementary material for: Genome-wide analysis identifies Homothorax and Extradenticle as regulators of insulin in Drosophila Insulin-Producing cells
Source: PLoS Genet. 2022 Sep 12;18(9):e1010380. doi: 10.1371/journal.pgen.1010380 (PMC9499297; doi:10.1371/journal.pgen.1010380)
Supplement: Attachment — Submitted filename: Winant et al_Response to reviewers.docx [file pgen.1010380.s014.docx]

Reviewer #1: This is an interesting study by Winant and colleagues analyzing on a broad

level the inputs regulating dILP2,3,5 expression in Drosophila. The study identifies and

focuses on Homothorax as a transcription factor regulating dILP expression. These dILPs are the fly equivalents of human insulin and insulin-like growth factor, and hence play an

important role in fly development and physiology. Hence understanding their regulation is

interesting and will be useful for a wide range of people working on fly development.

The main finding of this manuscript is the identification of Hth as a transcription factor

regulating dILP expression. Essentially, Hth knockdown causes reduced dILP protein levels. It would be good to clarify if this is a direct or indirect regulation. Are there Hth binding sites

near the dILP promoters or in an enhancer region? PMID 21483667 seems to have done a

genome-wide ChIP for Hth - is there any useful information there? Can an enhancer be

identified that is Hth responsive?

**We thank the reviewer for these comments and the suggestion to determine whether Hth directly regulates dilp2, 3 and 5 or not. To do so, we scanned *dilp2*, *-3* and *-5* enhancers for the presence of Hth binding motifs using bioinformatic tools. Indeed, we identified 2, 3 and 3 Hth binding sites in *dilp2, -3* and *-5* enhancers, respectively. (p<0.001, FIMO, MEME suite, included in Figure S9). This shows that direct regulation of the dilp2, -3 and -5 by Hth is a distinct possibility that can be further explored experimentally. The genome-wide ChIP for Hth (in PMID 21483667)** **was not informative for our study as this was focused on haltere imaginal discs and 0-16h embryos.**

1. Fig 2B - it is hard to see, given such a good correlation, which genes are the dimorphic ones. It would be helpful if the dots in panel B could be labeled with the same colours as the ones used in panel B' depending on whether they are significant in one sex only, or in both sexes. (Presumably the ones further from the diagonal will be dimorphic - ie light grey?)

**We thank the reviewer for this suggestion and have modified the figure as requested.**

2. Fig 3 - reduced IPC immunostaining for the dILPs can either be due to reduced expression or increased secretion. Since Hth was identified in a Q-PCR screen for genes that affect dILP expression at the mRNA level, the phenotype seen in Fig 3 is most likely reduced expression. It would be helpful to the reader to show the Q-RT-PCR results as a graph in Fig 3 (similar to Fig 4C-E for exd) This cannot be appreciated so easily and qualitatively in the heat map in Fig 1C.

**We have included the qPCR results for *Hth^RNAi^* in Figure 3 as Panel D, F and H.**

3. Why is there no effect of Hth knockdown in the IPCs on body weight? Shouldn’t such a dramatic drop in dILP2 expression cause a size effect?

**We agree with the reviewer that given the prominent effect of hth depletion on *dilp2* expression that a size effect would be likely. In our initial submission we saw a non-significant reduction in weight. Stimulated by the reviewer's comments, we repeated our previous weight experiments with an increased sample size (n>20) and in more controlled conditions. Our previous experiment used CyO siblings (deriving from the same food vial) as an internal control. This has the advantage of not having to worry about any differences in larval density and possible crowding effects. In the revised version, we collected early L1 larvae and placed them in a fixed density in food vials (20 larvae per vial), thus controlling for any confounding effects of high larval density on size phenotypes. We also measured additional size parameters such as head size and wing area. These results consistently show the presence of a size phenotype caused by *hth* knockdown in the IPCs. We have included these results in figure 5. Knocking down *exd* on the other hand did not yield any significant size phenotypes. As adult size is determined in the larval stage, we elaborate on these differences between Exd and Hth in the larval stage in figures 6, S6, and S7.**

Fig 3 - In dilp2-GAL4>Hth-RNAi animals, why does dILP2 expression drop so much while dILP2-GAL4 still works and drives expression of GFP? (ie why is there a discrepancy between expression of the endogenous dILP2 and the dILP2-GAL4?)

**We thank the reviewer for raising this comment. Given the role of Hth in regulating *dilp2* expression, we agree that knocking down Hth could have an effect on GAL4 driver strength. To address this, we measured GAL4 driver strength by measuring GFP intensity in the IPCs of *Dilp2-GAL4>nlsGFP, hthRNAi* brains and compared this to *Dilp2-GAL4>nlsGFP, mcherryRNAi* controls. Here we see a reduction of roughly ~40% GFP intensity, indicative of reduced driver strength. This relatively mild reduction in GAL4 activity, compared to the strong dilp2 expression effect, could be explained by the amplification effect of GAL4 which might minimise the effect. The different genomic context of the GAL4 insertion (chr2 as opposed to chr3 for the *dilp2* locus) could also explain why the effect on GAL4 activity is comparably smaller.**

**We nevertheless checked with a second GAL4 line whether we could recapitulate the observed dilp2-mediated phenotypes. We used OK107-GAL4, which expresses GAL4 under control of the Ey regulatory sequences that are not regulated by Hth (Adachi et al 2003, Mech Dev; Clements et al 2009, Dev Biol - see also figure S8 where we show that Ey levels are unaltered by *hth* knockdown in the IPCs). These flies show similar reductions in *dilp2* mRNA levels (18% of controls in males and 7% of controls in females with *OK107-GAL4* compared to 11% of controls in males and 15% of controls in females using *Dilp2-GAL4*). These flies also show similar effects on adult weight. These results thus show that the observed phenotypes are not significantly affected by the self-limiting nature of *Dilp2-GAL4>hthRNAi*. These results are included in figure S2.**

Reviewer #2: The manuscript by Winant and colleagues describes the interesting finding that the Homothorax-Extradenticle (Hth-Exd) complex regulates the expression of several insulin like peptides (ILPs) within the Insulin Producing Cells (IPCs) of Drosophila. The authors identified the Hth-Exd complex by analyzing the expression of Dilp genes within nearly 200 inbred fly lines. These lines are sequenced so gene variants can quickly be correlated with changes in expression. Of the 57 genes that the authors were able to interrogate through an RNAi screen, Hth and Exd had some of the strongest effects on Dilp expression. The authors go on to show that unlike the situation in the mammalian pancreas, the Hth-Exd complex does not regulate Pax6 transcription factors within the IPCs of Drosophila. The authors also show that several genes of the retinal determination network of which Hth-Exd and Pax6 are members, are expressed within the IPCs of the fly. And lastly, they show that depletion of the RD network members affects the expression of Dilps. A lot of work has gone into this manuscript and the data are quite convincing. However, in its present form, I am not sure that the results advance our understanding of Dilp regulation to a sufficient degree to warrant publication in PLoS Genetics. I have a few suggestions that I hope the authors will consider for a revised submission.

**We thank the reviewer for these comments and suggestions. Below we have outlined the additional work that was done to address these comments and suggestions.**

I think that a detailed phenotypic characterization of what happens to the fly when Hth and Exd are removed just from the IPCs will really strengthen the manuscript. As it stands now, the authors have identified an upstream regulator but have not shown us what happens when Hth-Exd are removed.

**We agree that a more detailed characterization is valuable and have included a number of phenotypes associated with Exd and Hth depletion in Figure 5.**

**As such, in addition to the reduced fecundity and increased oxidative stress resistance phenotypes from the original submission (now in Figure 5 F - G), we also did a more detailed phenotyping of size defects upon Hth/Exd depletion (Figure 5 A-C) and looked for the presence of any developmental timing phenotypes (Figure 5 D). This revealed that Hth but not Exd is essential for determining adult size. Furthermore, we also measured total TAG content in flies, showing that knocking down hth results in increased total TAG levels (Figure 5 E).**

**As both adult size and developmental timing are dependent on insulin signalling during the larval stage, this more in-depth phenotyping revealed that there are significant differences in *dilp* regulation between Hth and Exd in larvae that are not obvious in the adult stage. We elaborate on these differences at the larval stage in Figures 6, S6, S7.**

It would be helpful if the authors could correlate the regulation of Dilps by Hth-Exd to environmental conditions. Under which conditions is the Hth-Exd activated in the IPCs (ie. Starvation, well fed, constitutive?).

**We thank the reviewer for this interesting suggestion. We attempted to correlate the activity of the Hth-Exd complex in different nutritional conditions with the nuclear localization of Exd and Hth. We hypothesised that different nutritional conditions could affect nuclear localization or Hth/Exd expression in the IPCs.**

**To test this, we placed adult flies on different food media (sucrose only, protein rich (Yeasted) and starvation medium) for 72h. We then assessed the presence of Hth and Exd in IPC nuclei via Immunohistochemistry. This revealed that nutritional status did not noticeably alter Hth/Exd in the IPCs.**

**If Hth/Exd are only necessary for *dilp* regulation in specific nutritional conditions (e.g. well fed or in the presence of sugar) then depleting *hth* or *exd* in conditions lacking these nutrients would result in the absence of any effects on *dilp* mRNA levels. To test this, we**

**subjected *Dilp2GAL4>hthRNAi* and > *exdRNAi* flies to these same nutritional conditions as above and measured *dilp2*, *-3* and *-5* mRNA levels. mRNA levels had almost identical effects compared to flies that were fed control food (with the exception of *dilp5* levels in sucrose only condition, where dilp5 is only non-significantly increased compared to controls). This suggests that the role of Hth/Exd in dilp regulation is nutrient independent and must therefore play a constitutive or developmental role in the IPCs. We included these results in Figure S5**

While it looks like the IPCs are present when the Hth-Exd complex is knocked down it would be helpful if the authors could provide a quantitative measure of how many IPCs are present in these knockdowns when compared to wild type. Changes in Dilp expression could be due to changes in IPC numbers – it will be helpful to see if the IPC numbers change or not. Similarly, I would suggest that the authors do the same for the other RD members. The results might strengthen their model or change it (which could be very interesting as well).

**We agree that the effects of reduced *dilp2* and *-5* could be explained by differences in IPC cell numbers. We therefore assessed IPC numbers by systematically counting GFP+ nuclei in the Pars intercerebralis of adult brains of *Dilp2GAL4;nlsGFP>RDGN-RNAi* flies. We used this approach previously in *Clements et al. 2021,* Frontiers in Endocrinology. This revealed that there were no significant effects on IPC cell number when any of the RDGN TFs were knocked down (Figure S10).**

What happens to Dilp expression if the levels of the Hth-Exd complex are elevated within the IPCs.

**Overexpression of Hth or Exd did not significantly affect *dilp2, -3* or *5* mRNA levels nor did it significantly alter adult weight. We included these results in Figure S4.**
